# Radiolabeling Method for Lyophilizate for Dry Powder Inhalation Formulations

**DOI:** 10.3390/pharmaceutics14040759

**Published:** 2022-03-31

**Authors:** Kahori Miyamoto, Tomomi Akita, Chikamasa Yamashita

**Affiliations:** Department of Pharmaceutics and Drug Delivery, Faculty of Pharmaceutical Sciences, Tokyo University of Science, 2641 Yamazaki, Noda, Chiba 278-8510, Japan; 3a17706@ed.tus.ac.jp (K.M.); akitat@rs.tus.ac.jp (T.A.)

**Keywords:** dry powder inhalation, radiolabeling, lung deposition

## Abstract

Human lung deposition data is non-mandatory for drug approval but very useful for the development of orally inhaled drug products. Lung deposition of inhaled drugs can be quantified by radionuclide imaging, for which one of the first considerations is the method used to radiolabel formulations. In this study, we report the development of a radiolabeling method for lyophilizate for dry powder inhalation (LDPI) formulations. TechneCoat^TM^ is one method that can radiolabel drug particles without using solvents. In this method, particles are radiolabeled with a dispersion of ^99m^Tc-labeled nanoparticles called Technegas^TM^. Because a LDPI formulation is not comprised of particles but is a lyophilized cake aerosolized by air impact, the TechneCoat method cannot be used for the radiolabeling of LDPI formulations. We therefore modified the TechneCoat apparatus so that LDPI formulations were not aerosolized by the Technegas flow. Radiolabeling using a modified TechneCoat apparatus was validated with model LDPI formulations of interferon alpha (IFN). IFN of ^99m^Tc-unlabeled, IFN of ^99m^Tc-labeled, and ^99m^Tc of ^99m^Tc-labeled LDPI formulations showed similar behavior, and differences from IFN of ^99m^Tc-unlabeled LDPI formulations were within ±15% in aerodynamic particle size distribution measurement. Our radiolabeling method for LDPI formulations may be useful for the quantification of drug deposition in human lungs.

## 1. Introduction

Pulmonary drug delivery in the form of dry powder inhaler (DPI) is attracting interest as a technique for both local and systemic application [1,2]. DPI is especially attractive for the systemic application of biopharmaceutics such as peptides and proteins because the lung provides an enormous surface area, a relatively low enzymatic milieu, and a rapid absorption route that bypasses first-pass metabolism [1,3,4,5]. We have studied a lyophilizate for a dry powder inhalation (LDPI) system [6,7,8] and reported that a LDPI system can deliver a peptide systemically [9]. The LDPI system is quite a unique inhalation system in that the LDPI formulation, a lyophilized cake, is aerosolized just upon inhalation. Due to the simple manufacturing process of lyophilization, LDPI systems have advantages such as the fact that drugs can be formulated to avoid high temperature or shear stress [9,10], and LDPI formulations can ensure high content uniformity even when drug content is low [11]. We have developed a method to evaluate LDPI formulations using in vitro and in vivo models [9,12]. To put a LDPI system into practical application, the remaining task is the development of a method to measure lung deposition. Aerodynamic particle size distribution affects the lung deposition of inhaled drug, and lung deposition relates to drug efficacy and patient response [13,14,15,16,17]. Thus, human lung deposition data enable a seamless transition between in vitro testing in the laboratory and pivotal clinical studies of efficacy and safety.

The lung deposition of an inhaled drug can be quantified by radionuclide imaging, for which one of the first considerations is the method used to radiolabel the formulation. Thus, we aimed to develop a radiolabeling method for LDPI formulations that can be used to quantify drug deposition in human lungs. The radioisotope ^99m^Tc is most commonly used in the radiolabeling of drug formulations [18,19,20,21]. Among ^99m^Tc-labeling methods, we focused on TechneCoat^TM^ [22,23], which was developed for the dry labeling of DPI formulations. In the conventional radiolabeling method, ^99m^Tc-pertechnetate is added to the drug powder in an organic or aqueous solvent [19,24]. However, the use of a solvent may change the particle size distribution of a DPI formulation [23,25]. In the TechneCoat method, drug particles of DPI are radiolabeled with a dispersion of ^99m^Tc-labeled nanoparticles called Technegas^TM^ [26]. This dry labeling method can solve the above problem of the conventional method, but there is another problem in labeling LDPI formulations. Technegas is formed by heating sodium pertechnetate in a carbon crucible and is drawn out through drug particles by a vacuum pump. The particles are labeled by the Technegas stream. It is difficult to apply TechneCoat with no change to the LDPI system because the LDPI formulation consists not of particles but of a lyophilized cake that is aerosolized by air impact. Therefore, we modified the TechneCoat apparatus so that LDPI formulations are not aerosolized by the Technegas stream.

From the above, we decided to develop a ^99m^Tc radiolabeling method for LDPI formulations using the modified TechneCoat apparatus in an effort to quantify the drug deposition in human lungs. In this study, we used LDPI formulations of interferon alpha (IFN) as model formulations because the LDPI system is expected to be used for the systemic application of biopharmaceutics such as peptides and proteins, and IFN is comparatively easy to obtain and to measure by commercial assay kit.

## 2. Materials and Methods

### 2.1. Materials

IFN, L-phenylalanine (Phe), sodium pertechnetate, isotonic sodium chloride solution, Eagle’s minimal essential medium (MEM), l-glutamine, sodium bicarbonate, and bovine serum (BS) were all purchased from Sigma-Aldrich (St. Louis, MO, USA). Glass vials on which alkali-elution prevention processing has been performed (2 mL, φ18 mm, height 28 mm) were purchased from Iwata Glass Industrial (Osaka, Japan). Rubber stoppers (F-15) were purchased from NIPRO (Osaka, Japan).

### 2.2. Preparation of Freeze-Dried Cake of IFN

A solution containing 80,000,000 IU/mL of IFN with 4 mg/mL of Phe was prepared. Then, 500 µL of the prepared solution was dispensed into each glass vial and lyophilized with a freeze dryer (Triomaster II, Kyowa Vacuum Engineering, Tokyo, Japan). The solution was frozen by holding shelf temperature at −50 °C for 3 h. For primary drying, shelf temperature was ramped at 0.06 °C/min and held at −30 °C for 6 h at a pressure of 20 Pa. For secondary drying, shelf temperature was ramped at 0.33 °C/min to 30 °C and then ramped at −0.08 °C/min and held at 25 °C for 0.5 h.

### 2.3. Radiolabeling of Freeze-Dried Cake by Modified TechneCoat Appartus

**Figure 1:**
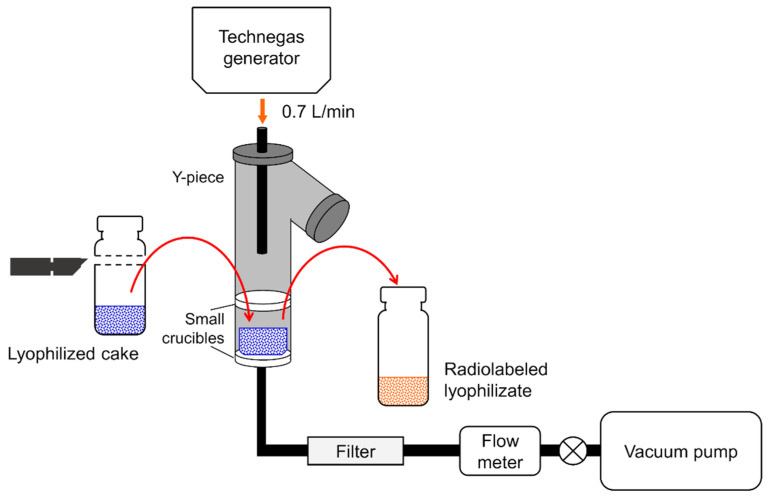
Method for radiolabeling of freeze-dried cake by modified TechneCoat apparatus. The apparatus consists of two small crucibles and a Y-piece. The lyophilized cake is recovered from the vial and placed onto the sinter of the bottom crucible. The top crucible is then sealed on top of this, and the Y-piece (with one end sealed and one end having a Technegas connection) is added. Technegas (produced using 1 simmer) is pulled through the apparatus at 0.7 L/min using a vacuum pump for 10 min, with the flow rate being monitored and controlled with the flow meter and valve.

The TechneCoat apparatus (Pharmaceutical Profiles Ltd., Nottingham, UK) is a device developed for the physical adsorption of ^99m^Tc to conventional inhalant forms of drugs with particle sizes of ≤5 μm adsorbed on lactose. LDPI, on the other hand, is a DPI in which the lyophilized cake is instantly microparticulated by the impact of air introduced during inhalation. Therefore, in order to physically adsorb ^99m^Tc onto LDPI, it is necessary to radiolabel ^99m^Tc into the matrix structure of the lyophilized cake while the lyophilized cake is not disintegrating. The usual TechneCoat apparatus uses 1.0 L/min as the flow rate of Technegas, but at this flow rate, the lyophilized cake collapses and ^99m^Tc could not be physically adsorbed on the lyophilized cake. Therefore, to prevent the collapse of the lyophilized cake, the flow rate of Technegas was reduced to 0.7 L/min, and to ensure that Technegas flowed evenly throughout the lyophilized cake, as shown in Figure 1, a glass filter was placed on the top and bottom of the cake. By modifying the TechneCoat apparatus in this way, we succeeded in physically adsorbing ^99m^Tc on LDPI while maintaining the features of LDPI.

A cotton wool plug was placed into a vial using tweezers, and then the vial was broken using a pipe cutter. The cotton wool plug was removed using tweezers with the vial upside down to prevent glass contamination of the lyophilizate. Then, the freeze-dried cake was recovered from the vial and was radiolabeled using the modified TechneCoat apparatus (Figure 1). Technegas (produced using 1 simmer) was pulled through the apparatus at 0.7 L/min using a vacuum pump for 10 min. After 10 min, the lyophilizate was transferred into an empty vial. The amount of radioactivity used varied between 1 and 13 MBq.

### 2.4. Quantification of IFN by Immunoassay

IFN was measured by enzyme-linked immunosorbent assay using a VeriKine Human IFN-α ELISA kit (R&D Systems, Minneapolis, MN, USA).

### 2.5. Counting Method for Radiolabeling of ^99m^Tc

The level of radioactivity was measured using a gamma camera (110, Technicare, Cleveland, OH, USA) fitted with a low-energy collimator on the ^99m^Tc channel.

### 2.6. Emitted Dose Test for LDPI Formulations of IFN

An emitted dose (ED) test was performed based on the uniformity of delivered dose test as described for inhalation powders in the European Pharmacopoeia (EP). The LDPI formulation including the device (Otsuka, Tokyo, Japan) was connected to the ED apparatus by means of an inlet adaptor. Air was drawn down through the apparatus at a set flow rate of 50 L/min with pressure drop at 4 kPa using a vacuum pump (HCP5, Copley Scientific Limited, Nottingham, UK) for 4.8 s with the flow rate being monitored and controlled with a critical flow controller (TPK2000), and flow meter (DFM2000) (both from Copley Scientific Limited). After a single dose was discharged, the apparatus was washed out with 5% BS-MEM solution for quantification of IFN. The test was repeated in triplicate. ED% was defined as the proportion of IFN content collected from the ED apparatus to IFN content of the formulations.

### 2.7. Measurement Method for Aerodynamic Particle Size Distribution of IFN and Radiolabel

Aerodynamic particle size distribution of LDPI formulations was measured according to the EP <2.9.18> Apparatus C—Multi-stage liquid impinger (MSLI) procedure for powder inhalers. As with the ED test, air was drawn down through the apparatus at a set flow rate of 50 L/min with pressure drop at 4 kPa for 4.8 s. Preparation of sample solutions for IFN determination was also performed in the same way as for the ED test. When measuring radioactivity level, apparatus imaging was performed before rinsing out. The test was repeated in triplicate. Fine particle fraction (FPF) was defined as the proportion of fine particle dose (FPD) to ED. FPD, with a mass of IFN of less than 5 μm, was calculated according to the EP. Effective cut-off diameters for stages 2, 3, and 4 were 14.2, 7.4, and 3.4 μm, respectively, at a flow rate of 50 L/min. MSLI stages are grouped into the following four stages: (1) Vial, Device, and Induction port; (2) Stages 1 and 2; (3) Stage 3; (4) Stages 4 and 5. The ratio to unlabeled drug of labeled drug or radiolabel in each group was calculated.

## 3. Results

### 3.1. Validation of the Lyophilizate Transfer Process

**Figure 2:**
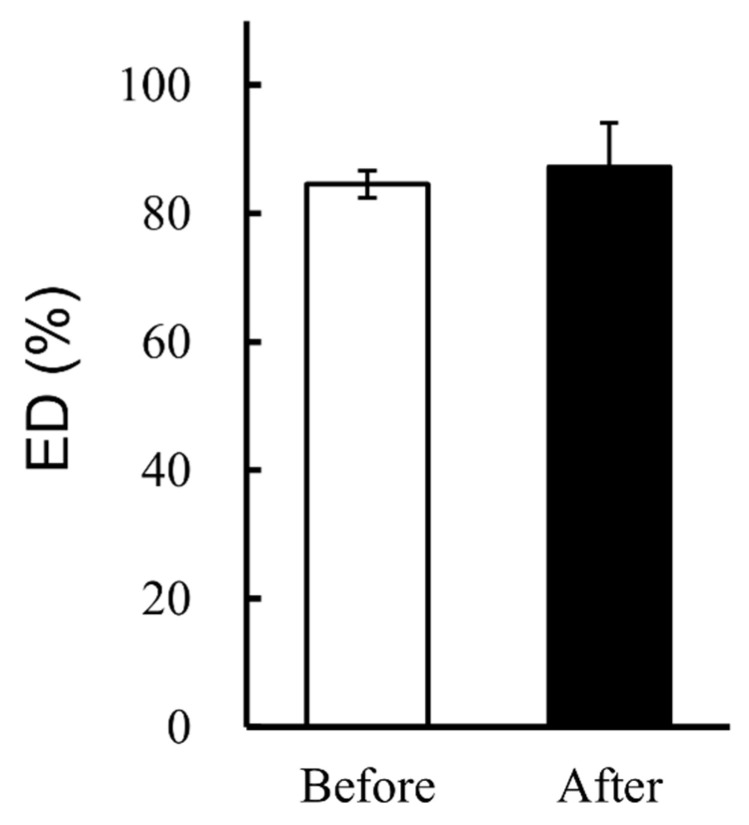
Results of emitted dose (ED) test for ^99m^Tc-unlabeled LDPI formulations before and after the transfer process (mean ± SE, *n* = 3). ED% of LDPI formulations containing 40,000,000 IU/vial of IFN showed no significant change when the lyophilized cake was transferred into another vial.

**Figure 3:**
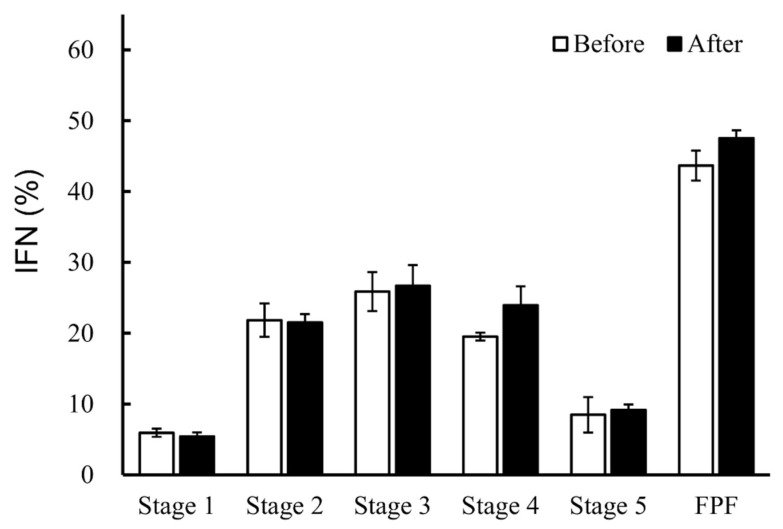
Aerodynamic particle size distribution of ^99m^Tc-unlabeled LDPI formulations before and after the transfer process (mean ± SE, *n* = 3). No significant change in aerodynamic particle size distributions of the LDPI formulations containing 40,000,000 IU/vial of IFN was shown when the lyophilized cake was transferred into another vial.

First, we confirmed that the lyophilizate transfer process does not affect the performance of the LDPI formulations. The lyophilized cake was transferred according to the radiolabeling method described in Section 2.3 but was not labeled. Then, the ED test and measurement of aerodynamic particle size distribution were performed. The results of the emitted test for intact and transferred cake were very similar (Figure 2). The aerodynamic particle size distributions of intact and transferred cake were also very similar (Figure 3). These data indicate that the process of transferring the lyophilized cake does not adversely affect the performance of the LDPI formulations.

### 3.2. Validation of Radiolabeling Method for LDPI Formulations

Since IFN cannot be measured during gamma scintigraphy performed on humans, measuring ^99m^Tc as a surrogate for IFN with a gamma camera is needed. To use ^99m^Tc as a surrogate marker, it is also important that the behavior of IFN measured by ELISA in ^99m^Tc-unlabeled and ^99m^Tc-labeled LDPI formulations match. Therefore, we compared the three formulations of IFN of ^99m^Tc-unlabeled LDPI formulations, IFN of ^99m^Tc-labeled LDPI formulations, and ^99m^Tc of ^99m^Tc-labeled LDPI formulations, and confirmed whether the intrapulmonary distribution could be estimated using ^99m^Tc as a surrogate marker.

**Figure 4:**
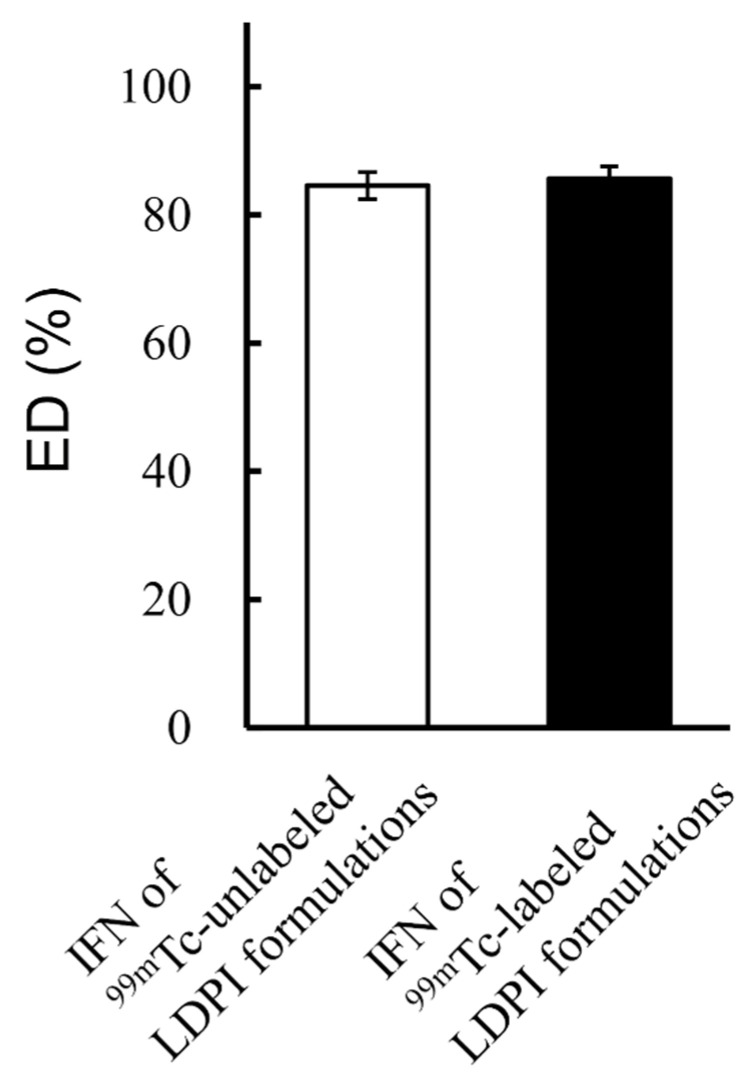
Results of emitted dose (ED) test for IFN of ^99m^Tc-unlabeled and labeled LDPI formulations (mean ± SE, *n* = 3). ED% of LDPI formulations containing 40,000,000 IU/vial of IFN showed no significant change when the lyophilized cake was radiolabeled. IFN of ^99m^Tc-unlabeled LDPI formulations are LDPIs that are unlabeled with ^99m^Tc, and IFN was measured by ELISA. IFN of ^99m^Tc-labeled LDPI formulations are LDPIs that are labeled with ^99m^Tc and IFN was measured by ELISA.

**Figure 5:**
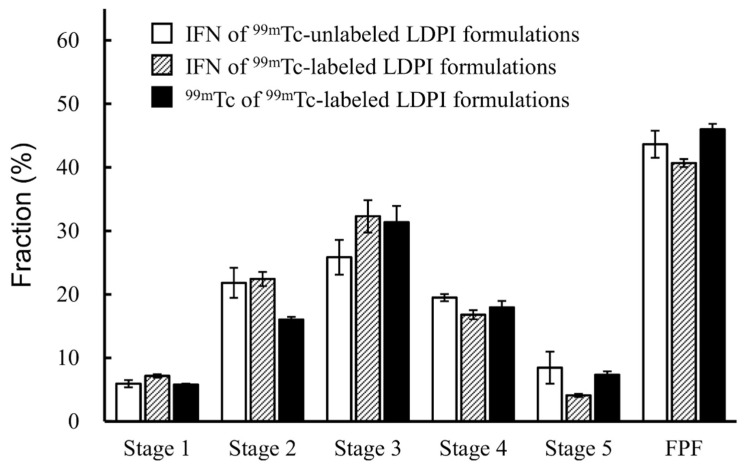
Aerodynamic particle size distribution of IFN of ^99m^Tc-unlabeled LDPI formulations, IFN of ^99m^Tc-labeled LDPI formulations, and ^99m^Tc of ^99m^Tc-labeled LDPI formulations (mean ± SE, *n* = 3). IFN of ^99m^Tc-unlabeled LDPI formulations, IFN of ^99m^Tc-labeled LDPI formulations, and ^99m^Tc of ^99m^Tc-labeled LDPI formulations showed similar aerodynamic particle size distribution. LDPI formulations containing 40,000,000 IU/vial of IFN were used. IFN of ^99m^Tc-unlabeled LDPI formulations are LDPIs that are unlabeled with ^99m^Tc, and IFN was measured by ELISA. IFN of ^99m^Tc-labeled LDPI formulations are LDPIs that are labeled with ^99m^Tc and IFN was measured by ELISA. ^99m^Tc of ^99m^Tc-labeled LDPI formulations are LDPIs that are labeled with ^99m^Tc and ^99m^Tc was measured by gamma camera as a surrogate for IFN.

The performance of LDPI formulations was compared between IFN of ^99m^Tc-unlabeled and labeled LDPI formulations. There was no significant difference in the results of the ED test between IFN of ^99m^Tc-unlabeled and labeled LDPI formulations (Figure 4). The aerodynamic particle size distributions of IFN of ^99m^Tc-unlabeled, IFN of ^99m^Tc-labeled, and ^99m^Tc of ^99m^Tc-labeled LDPI formulations were very similar (Figure 5). The ^99m^Tc of ^99m^Tc-labeled LDPI formulations slightly underestimated drug deposition in stage 2, but the difference was quite small and was not considered significant.

**Figure 6:**
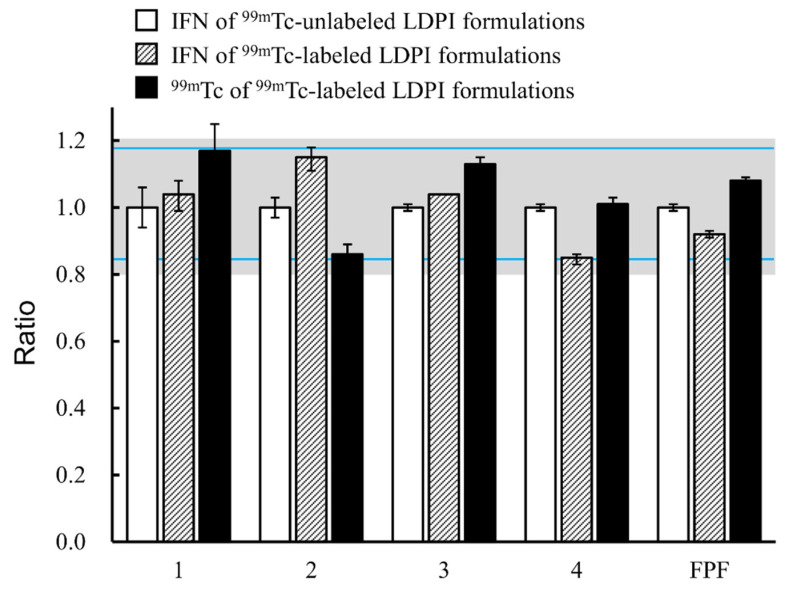
Ratio to IFN of ^99m^Tc-unlabeled LDPI formulations of IFN of ^99m^Tc-labeled LDPI formulations or ^99m^Tc of ^99m^Tc-labeled LDPI formulations per group of MSLI stages (mean ± 90% CI, *n* = 3). The mean ratio of IFN of ^99m^Tc-labeled LDPI formulations or the ^99m^Tc of ^99m^Tc-labeled LDPI formulations per group of MSLI stages to the IFN of ^99m^Tc-unlabeled LDPI formulations was within the range of 0.85–1.18. LDPI formulations containing 40,000,000 IU/vial of IFN were used. The gray box indicates the range 0.8–1.2, which has been used as the acceptance criteria for radiolabeling methods. The blue line indicates the range 0.85–1.18, which was recently proposed as the acceptance criteria. Group 1: Vial, device, and induction port, 2: Stages 1 and 2, 3: Stage 3, 4: Stages 4 and 5. IFN of ^99m^Tc-unlabeled LDPI formulations are LDPIs that are unlabeled with ^99m^Tc, and IFN was measured by ELISA. IFN of ^99m^Tc-labeled LDPI formulations are LDPIs that are labeled with ^99m^Tc and IFN was measured by ELISA. ^99m^Tc of ^99m^Tc-labeled LDPI formulations are LDPIs that are labeled with ^99m^Tc and ^99m^Tc was measured by gamma camera as a surrogate for IFN.

The mean ratio of IFN of ^99m^Tc-labeled or the ^99m^Tc of ^99m^Tc-labeled LDPI formulations per group of MSLI stages to the IFN of ^99m^Tc-unlabeled LDPI formulations is shown in Figure 6. The mean ratio was within the range 0.85–1.18. Moreover, 90% confidence intervals (CI) ranged roughly from 0.85 to 1.18. Only the 90% CI of the ^99m^Tc of ^99m^Tc-labeled LDPI formulations in group 1 was slightly over 1.18. Although the radiolabel in group 1 may have been overestimated, IFN of ^99m^Tc-labeled or ^99m^Tc of ^99m^Tc-labeled LDPI formulations deposited in each part of group 1 was low, and the absolute difference in the proportion between IFN of ^99m^Tc-unlabeled and ^99m^Tc of ^99m^Tc-labeled LDPI formulations was sufficiently small (within ±2%). In addition, group 1, comprising the sum of vial, device, and induction port, is not important for lung deposition measurement because the proportion in group 1 corresponds to extrapulmonary parts. These data indicate that the radiolabeling process does not adversely affect the performance of the LDPI formulations. It became clear that ^99m^Tc can be used as a surrogate marker.

## 4. Discussion

Human lung deposition data is non-mandatory for drug approval but very useful for the development of DPI. These data enable a seamless transition between in vitro testing in the laboratory and pivotal clinical studies of efficacy and safety [15]. Lung deposition data can also be used for the comparison between formulations with different inhalation devices [27]. The radionuclide imaging method is commonly used to determine the lung deposition of an inhaled drug, and when the method is performed, the radiolabeling method also needs to be considered. The requirement for the radiolabeling method of DPI formulations is that the un-radiolabeled formulation which measures the main drug at the time of evaluation, the radiolabeled formulation which measures the main drug at the time of evaluation, and the radiolabeled formulation which measured the radiolabel as a surrogate marker, show the same behavior.

Prior to the comparison of drug behavior, we validated the transfer process of the lyophilized cake. DPI formulations are commonly prepared as particles and particles are filled in a container. However, the LDPI formulation is a lyophilized cake within the vial itself, and the lyophilizate is aerosolized by convection flow of air in the vial just upon inhalation. Hence, there was a concern that the transfer process might change the aerosolization performance of LDPI formulations. However, no significant differences in ED and aerodynamic particle size distribution were shown between the intact and transferred lyophilizate (Figure 2 and Figure 3). The effect of the lyophilizate transfer process on the aerosolization performance of LDPI formulations was considered to be negligible.

Then, the labeling process by Technegas stream was validated. The acceptance criterion for radiolabeling methods that the FPF ratio of radiolabel to unlabeled drug should be within the range 0.8–1.2 was used [18,20,28]. LDPI formulations radiolabeled by our method using the modified TechneCoat apparatus met the above criterion (Figure 6). More strict criteria are recommended in some publications, including the European Medicines Agency (EMA) guideline [18,29,30]. For example, Devadason et al. [18] proposed the following acceptance criteria: If regional lung deposition is to be quantified, the mean ratio of radiolabeled drug/the radiotracer per impactor stage, or group of impactor stages, to the reference drug should be within 0.85–1.18. As shown in Figure 6, the LDPI formulations radiolabeled by our method also met these strict criteria. Moreover, 90% CIs ranged roughly from 0.85 to 1.18 except for 99 mTc of 99 mTc-labeled LDPI formulations group 1. The 90% CI of ^99m^Tc of ^99m^Tc-labeled LDPI formulations group 1 was slightly over 1.18, but the difference between IFN of ^99m^Tc-unlabeled and ^99m^Tc of ^99m^Tc-labeled LDPI formulations in group 1 was considered to be negligibly small. From the above, we considered that IFN of ^99m^Tc-labeled LDPI formulations were equivalent to IFN of ^99m^Tc-unlabeled LDPI formulations and that the aerodynamic particle size distributions of the drug and ^99m^Tc of ^99m^Tc-labeled LDPI formulations were equivalent.

Whether the radioactivity is sufficient for use in a clinical study also requires consideration. Because LDPI formulations are aerosolized by air impact, the flow rate of Technegas in our radiolabeling method is very low so that the lyophilizate is not aerosolized by the Technegas stream. The amount of radioactivity used in this study varied between 1 and 13 MBq, and the range of radioactivity used in a clinical study would be 1 to 10 MBq [23,31,32,33]. It is thus expected that the radioactivity obtained by our radiolabeling method is adequate to perform clinical studies involving lung drug deposition.

## 5. Conclusions

We developed a radiolabeling method for LDPI formulations using a modified TechneCoat apparatus in this study. IFN of ^99m^Tc-unlabeled LDPI formulations, IFN of ^99m^Tc-labeled LDPI formulations, and ^99m^Tc of ^99m^Tc-labeled LDPI formulations showed quite similar aerodynamic particle size distributions. Differences in each IFN of ^99m^Tc-labeled LDPI formulation and ^99m^Tc of ^99m^Tc-labeled LDPI formulations from IFN of ^99m^Tc-unlabeled LDPI formulations were within ±15%, the acceptance criteria stated in the EMA guideline. It is expected that our radiolabeling method for LDPI formulations can be used for the quantification of drug deposition in human lungs.

## Data Availability

The data presented in this study are available on request from the corresponding author.
